# Multiple Myeloma and Renal Failure: Mechanisms, Diagnosis, and Management

**DOI:** 10.7759/cureus.22585

**Published:** 2022-02-25

**Authors:** Sumana Kundu, Surajkumar B Jha, Ana P Rivera, Gabriela V Flores Monar, Hamza Islam, Sri Madhurima Puttagunta, Rabia Islam, Ibrahim Sange

**Affiliations:** 1 Research, Radha Govinda Kar Medical College, Kolkata, IND; 2 Research, School of Medicine/Jinan University, Guangzhou, CHN; 3 Research, Universidad Americana (UAM) Facultad de Medicina, Managua, NIC; 4 Research, Universidad Central del Ecuador, Quito, ECU; 5 Research, Faisalabad Medical University, Faisalabad, PAK; 6 Research, Dr. Pinnamaneni Siddhartha Institute Medical College, Chinoutpalli, IND; 7 Research, Karamshi Jethabhai Somaiya Medical College, Hospital and Research Center, Mumbai, IND

**Keywords:** high cut-off dialyzer, dialysis, hematopoietic stem cell transplantation, chemotherapy, myeloma kidney, cast nephropathy, renal failure, multiple myeloma

## Abstract

Multiple myeloma (MM) is the second most common hematologic malignancy that involves monoclonal immunoglobulin (Ig)-producing plasma cells. Due to its multifaceted clinical manifestations and complications, it draws attention to various medical specialties like neurology, nephrology, orthopedics, cardiology, etc. Renal failure (RF) is one of the most common and most serious complications of MM that can be caused either by excess immunoglobulins that are nephrotoxic or some other causes like hypercalcemia, infection, etc. In this review article, we have discussed the pathogenesis of RF in MM, described the different diagnostic tools to diagnose RF in MM, and explained different treatment modalities to treat RF in MM, including certain general measures (i.e., hydration, withholding any nephrotoxic agents), renal replacement therapy, serum free light chain (SFLC) removal by plasma exchange and high cut-off dialyzer (HCO-HD), chemotherapy, hematopoietic stem cell transplantation (HSCT), and renal transplantation.

## Introduction and background

Multiple myeloma (MM) is a hematologic malignancy characterized by the pernicious proliferation of monoclonal plasma cells that destroy the bone marrow (BM) microenvironment and result in overproduction of monoclonal immunoglobulins (Ig), immunodeficiency, and osteoclast hyperactivation [1-3]. After Non-Hodgkin lymphoma, MM is the second most common hematologic malignancy, and it contributed to around 15% of hematologic malignancies diagnosed in 2010 [4]. MM is more common in men than in women, and the incidence is twice as high in African Americans as in Caucasians [2,5]. MM is usually a disease of the older population with 65 years being the median age for diagnosis, with less than 3% of patients presenting earlier than 40 years [1]. The potential risk factors for developing MM are obesity, ionizing radiation exposure, chronic antigen stimulation, genetics, and environmental exposures like certain occupations, i.e., farming and agriculture [5-8]. Genetic mutations like t (4;14), t (14;16), t (14;20), deletion 17p, gain 1q, or p53 mutation are associated with development of high-risk MM [9]. MM is a multistep process that involves the initial formation of a plasma cell clone and then malignant transformation of that precursor into plasma cell myeloma that is thought to be the final malignant stage of the disease process, which starts from a relatively benign disease called monoclonal gammopathy of undetermined significance (MGUS) [5,10]. The disease presents with anemia, recurrent infections, renal insufficiency, osteolytic bone lesions, and hypercalcemia [1,2,10]. MM is suspected when the serum paraprotein rises above 30 g/L on serum electrophoresis and is confirmed by a BM plasmacytosis of more than 10% on BM biopsy [10]. Treatment of MM includes chemotherapy and hematopoietic stem cell transplantation (HSCT), and most commonly used classes of drugs are-alkylating agents (melphalan, cyclophosphamide), proteasome inhibitors (bortezomib, carfilzomib), immunomodulatory drugs (IMiDs) (thalidomide, lenalidomide), and corticosteroids (dexamethasone, prednisone) [11]. MM is a multifaceted disease, and about 25%-75% of patients of MM develop renal failure (RF) from the disease itself or other unrelated causes. RF was the second most common cause of death (after infection) among the patients of MM, before the era of unlimited access to dialysis [12]. This review article aims to explore the pathogenic relationship between MM and RF with an emphasis on the diagnosis and management options for the same.

## Review

Pathogenic relationship between MM and renal failure

Although the major reason for developing RF in MM is the overproduction of nephrotoxic Ig, to a great extent, several non-Ig-related causes too can contribute [13,14]. Among the Ig-related causes, the most common cause was cast nephropathy (40-63%), followed by light chain deposition disease (LCDD) (20-25%) and amyloidosis (15-35%), as observed in the studies conducted on MM patients with renal impairment (RI) undergoing renal biopsies [12,15]. Where cast nephropathy has not been found to have any association with a predominant LC, LCDD is more frequently associated with the kappa (κ) LC and amyloid kidney with the lambda (λ) LC [16]. Cast nephropathy usually presents in more advanced stages of MM; however, this is not true for amyloid LC (AL)-amyloidosis or LCDD [15]. The nephrotoxicity of the LCs is determined by the degree of their self-aggregation and the decreased lysosomal degradation in the proximal tubular cells; therefore, their nephrotoxic potential is not always dependent on their concentration [17]. Common non-LC-related causes of RI in MM include hypercalcemia (the second most common cause of RF in MM after cast nephropathy), infections, hypovolemia (often associated with hypercalcemia), use of nephrotoxic drugs, use of contrast media, and renal amyloidosis [13,14].

**Figure 1:**
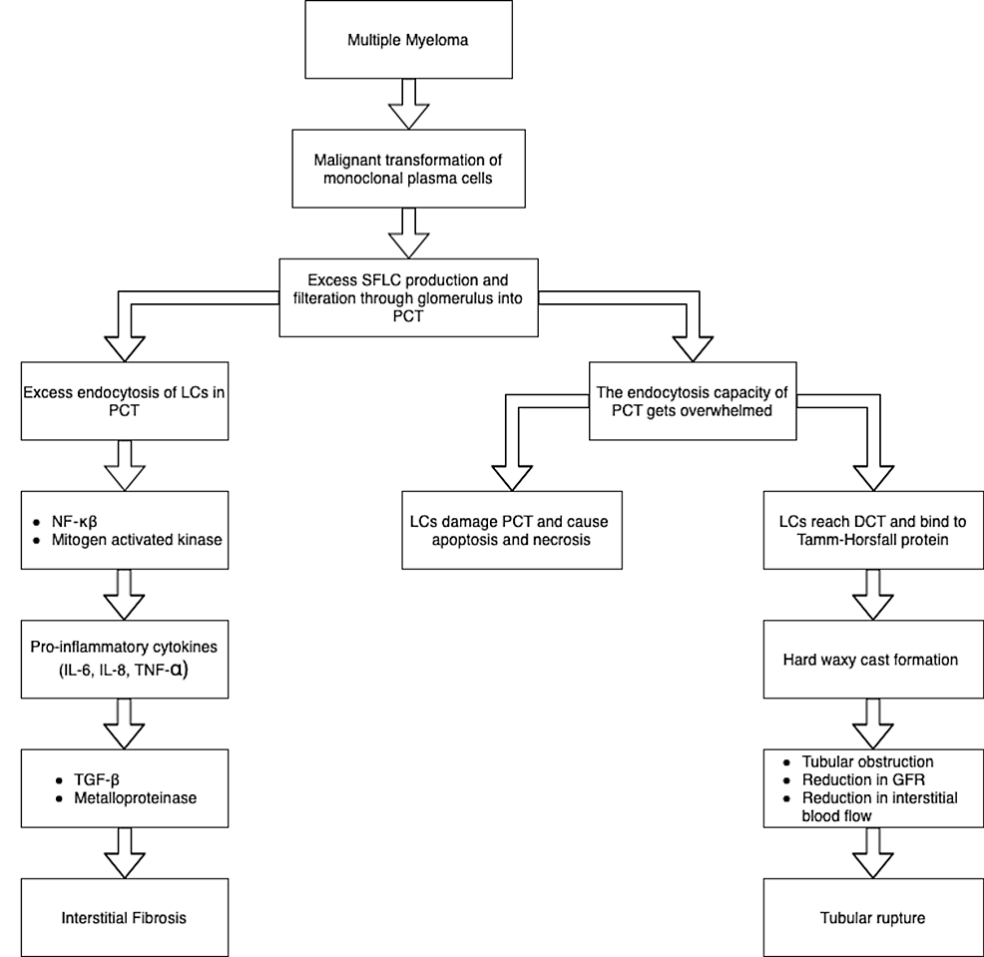
Pathogenesis of cast nephropathy SFLC: serum free light chain; PCT: proximal convoluted tubule; LCs: light chains; NF-κβ: nuclear factor- kappa beta; IL: interleukin; TNF-α: tumor necrosis factor- alpha; TGF-β: transforming growth factor-beta; DCT- distal convoluted tubule; GFR: glomerular filtration rate

Myeloma cast nephropathy, alternatively known as myeloma kidney is probably the most common and most important renal manifestation of MM [12]. This disorder is observed in more than 50% of patients who died from MM with renal involvement and in 40%-60% of renal biopsies performed in patients of MM with renal involvement [18-21]. The entity presents in more advanced stages of MM and has not been found to be associated with a predominant LC [12,17]. Almost all cases of myeloma kidney present with RF, and about two-thirds of the cases develop proteinuria [12]. Other manifestations of MM cast nephropathy are nephrogenic diabetes insipidus and rarely acquired adult Fanconi syndrome [13]. Normally, the excess serum free light chain (SFLC) gets filtered through the glomerulus and, on reaching the proximal tubule of the nephron, binds to the multiligand receptors including cubilin and megalin; after that, the formed complex gets endocytosed by the clathrin-dependent endosomal-lysosomal pathway in the proximal tubule cells [22-24]. However, when the SFLCs exceed the endocytosis capacity of the proximal tubule, then this excess SFLC causes damage to the proximal tubular cells resulting in apoptosis and necrosis [22-24]. Furthermore, the LCs enter the distal tubule, where they bind to the Tamm-Horsfall (TH) glycoprotein resulting in cast formation, and these casts obstruct renal tubules and reduce glomerular filtration and interstitial blood flow leading to tubular rupture, then, eventually interstitial nephritis (Figure 1) [22-25].

The rate of cast formation depends largely on the type and characteristics of LCs as well as specific features of TH protein (for example, the degree of its glycation) [12]. High concentrations of SFLC (usually higher than 1000 mg/dL), urinary acidification, dehydration, high urinary sodium and calcium, loop diuretics like furosemide, non-steroidal anti-inflammatory agents, intravenous contrast, hypercalcemia, and nephrogenic diabetes insipidus can potentiate cast nephropathy [12,22]. In addition, the overwhelmed endocytosis of LCs activates various proinflammatory cytokines and, through different mediators, causes interstitial fibrosis [26]. All these changes are seen on renal biopsy as a triad of injury in the proximal tubules, hard waxy casts in the distal tubules and interstitial inflammation and fibrosis (Figure 1) [13]. Myeloma cast nephropathy is associated with an exceptionally poor prognosis for both renal and overall survival (OS) [12]. However, if treated early, this entity can be potentially the most reversible of all the MM related renal diseases [13].

LCDD is rarely diagnosed on autopsy, whereas it is observed in 20% - 25% of patients with MM and renal involvements undergoing renal biopsy [12]. Patients with LCDD are younger (median age 58 years) than those with other types of renal involvement [12]. This entity is predominantly associated with the kappa LC [17]. Almost all cases of LCDD manifest with heavy proteinuria, and in most cases, elevated serum creatinine is diagnosed [12]. Initially, the glomerulopathic kappa LCs freely pass through the filtration membrane and get deposited in the subendothelial glomerular basement membrane (GBM), leading to submicroscopic GBM damage and selective proteinuria resembling minimal change disease [12]. Later, LCs reach the mesangial compartment and stimulate the proliferation of mesangial cells, resulting in proliferative glomerulonephritis (pattern of injury can be mesangial proliferative or membranoproliferative) [12]. With time, mesangial cells produce excess extracellular matrix (ECM) proteins (type four collagen, fibronectin, laminin, tenascin C) [12]. Concurrently, the activity of ECM catabolizing enzymes (like metalloproteinase 7) decreases, which additionally enhances the accumulation of ECM [12]. These two causes together lead to nodular glomerular sclerosis taking after Kimmelstiel-Wilson lesions observed in advanced diabetic nephropathy [27,28]. In all mentioned stages, proteinuria may be present, accompanied by a gradually decreasing glomerular filtration rate (GFR) [12]. On renal biopsy, the monoclonal proteins deposit along the glomerular and tubular basement membrane, mesangium and vessel walls and, by nature, these proteins are non-fibrillar and Congo red stain negative [27]. Immunofluorescence shows the fixation of monoclonal LC anti-sera along the basement membranes in 90% as linear deposits [13]. Under electron microscope, these are seen as granular deposits [27]. According to a study conducted by Pozzi et al., 35% of cases of LCDD were associated with extra-renal manifestations with commonly involved organs being the heart (21%) and the liver (19%) presenting as congestive cardiac failure, arrhythmia, and portal hypertension [29]. The lung, gastrointestinal tract, and neurological system are less frequently involved [13]. Nearly all patients with LCDD progress to rarely reversible RF [13].

Amyloidosis is found in less than 5% of the patients with MM on autopsy and in 15%-35% of renal biopsies performed in patients of MM with renal involvement [12]. Up to 80% of cases of amyloidosis present with proteinuria [12]. Amyloid deposition predominantly involves the glomeruli causing progressive RF with nephrotic syndrome [30]. However, in 10% of cases where the deposition is in the renal vasculature and tubulointerstitium, RF occurs without nephrotic syndrome [13]. A subcutaneous fat aspirate and BM biopsy can be diagnostic in 90% of cases, and if these are negative and the patient has RF, then a renal biopsy can be diagnostic in 95% of cases [30]. When stained with Congo red dye, the amyloid deposits produce apple-green birefringence under polarized light [30]. Specific anti-LC antibodies identify the type of amyloid [12]. Other manifestations can be neuropathy, orthostatic hypotension, hepatomegaly, cardiomyopathy and requires demonstration of amyloid in tissue [13]. Renal recovery is rare in this setting [13]. Survival of patients with AL-amyloidosis is significantly worse compared with that of patients with LCDD, and the prognosis is worsened mainly by extra-renal deposit [31].

Diagnosis of MM and RF

Serum protein and 24-hour urine protein electrophoresis and immunofixation have been used to diagnose and monitor disease; however, these tests are not always reported promptly, and in a patient with oliguric RF, 24-hour urine specimen is often difficult to obtain [13]. Furthermore, since the paraprotein band contains a complete Ig made up of two heavy chains associated with two LCs, in LC-only MM with RF and non-secretory disease, these tests are not helpful [32,33].

The SFLC assay is a same-day analysis, and it helps with the reclassification of non-secretory MM (owning to its ability to identify previously hidden determinants of LC) [33]. The reference range of the κ/λ ratio is 0.26-1.65, with a median ratio of 0.6 [34]. However, in the context of RF in MM, the SFLC ratio becomes 1.8 reflecting the overwhelmed SFLC clearing capacity of the reticuloendothelial system by the overproduction of the κ and λ producing plasma cells [34,35]. SFLC assay carries prognostic value, too [36]. For example, high SFLC levels at baseline indicate more aggressive disease and, therefore, poorer prognosis [37]. SFLC assay along with serum protein electrophoresis and immunofixation can accurately diagnose all myeloma cases without the need for urine collection [38].

β-2-microglobulin (B2M) is the LC of the major histocompatibility class 1 that is expressed on the surface of most nucleated cells [13,39]. Identification of the serum B2M is correlated with the MM tumor load, and a level more than 6 mg/L indicates poor prognosis [13,39]. However, B2M is also elevated in RF and therefore, in MM with RF, this system loses its usefulness [13,39].

Renal biopsy should be considered early on the course of the disease, particularly when serum creatinine rises above 30% of normal since it can be of great diagnostic and prognostic value [13,22]. For example, it is the renal histology that provides the initial diagnosis of MM in a patient presenting with RF and, several histological hallmarks, i.e., degree of renal fibrosis, strongly reflect the possibility of renal recovery [13,22]. Along with that, a renal biopsy can easily differentiate various histological patterns of kidney involvement in MM, which gives vital information regarding survival [15]. As per a study conducted by Montseny et al. on dialysis-dependent MM patients treated with chemotherapy, the median survival for cast nephropathy, LCDD, and AL-amyloidosis were 6, 18, and 48 months, respectively [15]. In addition, salient features on repeat biopsy also help to know treatment response, for instance, progression of signs of chronic damage (i.e. sclerosed glomeruli, interstitial fibrosis and edema, etc.), change in intratubular cast numbers and so on [22].

Management of renal failure in multiple myeloma

General Measures

RF in MM is a medical emergency; therefore, in this setting, various potential mechanisms of RF should be addressed simultaneously [40]. Non-steroidal anti-inflammatory agents, contrast dyes, loop diuretics, i.e., furosemide (because they may contribute to paraprotein cast formation), aminoglycosides, angiotensin-converting enzyme inhibitors, and angiotensin II receptor inhibitors, and other nephrotoxic agents should be avoided [22,41]. Hydration along with urinary alkalinization slightly decreases the concentration of the LCs and increases their solubility [13]. However, the benefit of urinary alkalinization is controversial since it can theoretically increase the risk of abnormal calcification in the kidney or other organs, especially in the presence of hypercalcemia [40]. Chemotherapy for MM should be started as soon as possible with drugs that are not excreted through kidney [13]. Hypercalcemia should be treated with aggressive hydration along with calcitonin, and bisphosphonates can be initiated once the RF improves [13,42]. In the case of tumor lysis syndrome (although it is rare in myeloma), rasburicase is remarkably effective; however, in refractory cases, allopurinol and hemodialysis should be considered [40].

Renal Replacement Therapy

In cases where hydration is not tolerated as well as cases where no improvement is found despite taking all the aforementioned general measures, renal replacement therapy or dialysis may be needed [13]. 88% of dialysis requiring MM patients receive hemodialysis, whereas only 12% have peritoneal dialysis, and although both are equally effective, the latter can increase the risk of bacterial peritonitis with long-term use [31]. Hemodialysis should be considered as an effective treatment method in serious complications of hypercalcemia (i.e., confusion, cardiac arrhythmias, etc.) as well as clinically significant tumor lysis syndrome refractory to rasburicase [40].

Plasmapheresis

Only 25% of total FLCs can be removed by plasma exchange over a 3-week period [14]. In addition, within a few hours after each pheresis session, rapid plasma refill occurs (owning to the fact that the LCs and IgG have high volume of distribution) [43,44]. That is why, the benefit of adding plasma exchange to chemotherapy in improving GFR, dialysis dependence or death is quite uncertain [45]. However, in patients who experience a rapid decline in FLC levels, renal outcomes are improved [46]. In a study conducted by Leung et al., patients who had 50% or more fall in SFLC recovered from dialysis [44]. Plasma exchange has a clear indication in hyperviscosity of plasma, usually seen with IgG3, IgM, and IgA myeloma [13,40].

High Cut-Off Dialyzer (HCO-HD)

A new generation of dialyzers with cartridges having pores larger than routine dialyzers can be used with extended dialysis to remove the SFLC [47]. One example is the Gambro HCO 1100 dialyzer (Dialysatoren GmbH, Hechingen, Germany), and one major downside of it is albumin loss (owing to its molecular cut-off that is similar to albumin) [47]. Extended hemodialysis with a HCO-HD for over a 3-week period will remove 90% of total FLCs [13]. As per an open-label study conducted by Hutchison et al. in 2009, where 19 patients with myeloma kidney (cast nephropathy) and dialysis-dependent acute RF were treated with the combination of chemotherapy and HCO-HD and 13 patients from the group who received uninterrupted chemotherapy, experienced sustained early reductions in SFLC concentrations becoming dialysis independent at a median of 27 days (range 13 to 120 days) [47]. Randomized controlled trials of this technique are underway both in Europe (European Trial of Free Light Chain Removal (EULITE)); and France (studies in patients with MM and RF due to cast nephropathy (MYRE)) [40].

Chemotherapy

**Figure 2:**
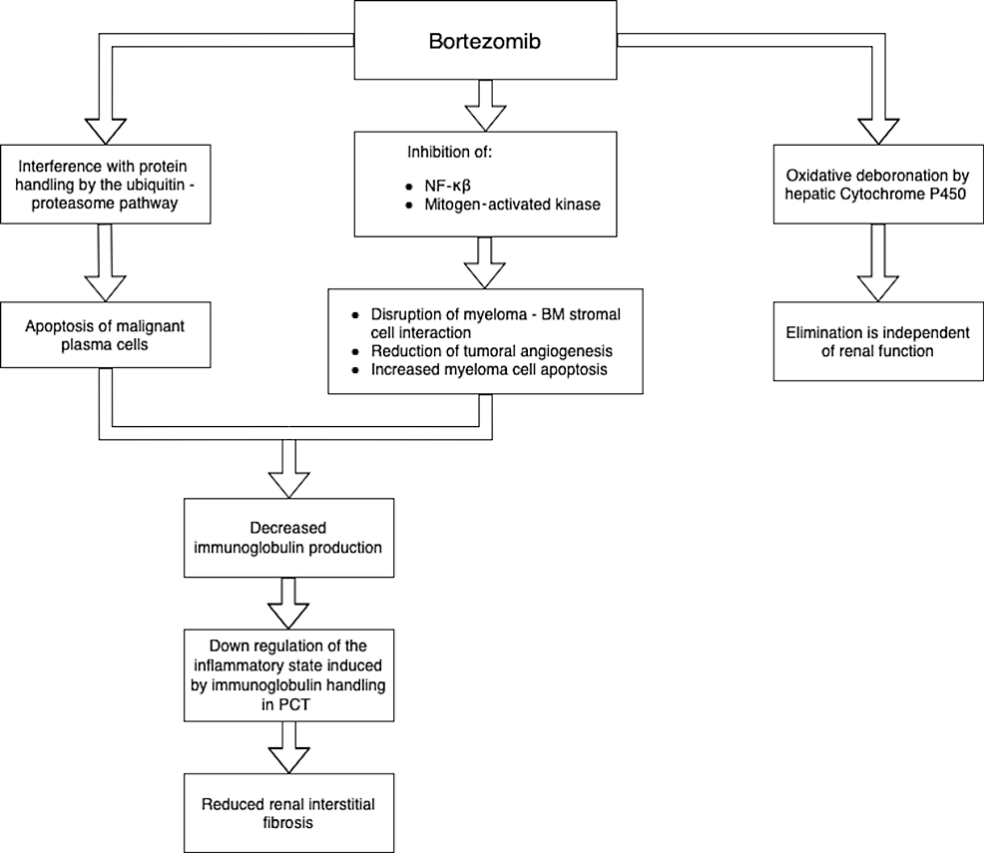
Mechanism of action of bortezomib NF-κβ: nuclear factor- kappa beta; BM: bone marrow; PCT: proximal convoluted tubule

Conventional chemotherapeutic regimens to treat MM with RF are VAD (vincristine, Adriamycin, dexamethasone), VCMP (vincristine, cyclophosphamide, melphalan, prednisone) [13]. When newly diagnosed patients with MM and RI were treated with melphalan and prednisone, they were affected by increased hematologic and infectious toxicity. Therefore, melphalan should be used in reduced doses in such patients [48-50]. Over the past decade, substantial improvement has been seen in treating MM patients with RF with the invention and usage of certain novel drugs like proteasome inhibitors (bortezomib) and IMiD (thalidomide and lenalidomide), which have demonstrated positive response both in the first-line treatment and relapsed cases of MM [14,51-53]. Bortezomib, a potent boronate peptide and a reversible proteasome inhibitor, is considered to be an important therapeutic step forward in the treatment of MM [14]. It acts through a multifaceted mechanism that is depicted in Figure 2 [54-57].

This drug’s rapid onset of action makes it effective for preventing permanent kidney injury [58-63]. Since this drug undergoes oxidative deboronation by hepatic cytochrome P450, its plasma elimination is not dependent on renal function; therefore, no dose reduction is required in RF patients [13].

Chanan-Khan et al. conducted a retrospective case analysis in 2006, where 24 patients with MM and advanced RF requiring dialysis support received bortezomib alone or bortezomib-based combination therapy and, among 20 patients with available response data, overall response rate (complete response (CR) + partial response (PR)) was 75%, with 30% CR + near CR (different types of responses are described in Table 1) [58,64].

**Table 1:** Different types of responses to anti-myeloma therapy CR: complete response; BM: bone marrow; LC: light chain; nCR: near complete response; PR: partial response; B-J proteinuria: Bence-Jones proteinuria

Type of response	Definition
CR	Disappearance of monoclonal proteins in serum and urine immunofixation analysis. BM aspirate is normal. BM biopsy with less than 1% LC restricted plasma cells on flow cytometry.
nCR	Positive immunofixation analysis of serum and urine. BM findings are normal.
PR	More or equal to 75% reduction from baseline serum monoclonal protein. Reduction to less than 100 mg/day in a case of B-J proteinuria. Normal BM aspirate and biopsy.

One patient was spared dialysis, and three other patients became independent of dialysis following bortezomib-based treatment, and these encouraging results suggest that bortezomib or bortezomib-based regimens can be used in MM patients requiring dialysis with manageable toxicities [58]. In 2011, Leal et al. conducted a dose-escalating and prospective pharmacologic phase I study sponsored by the US National Cancer Institute where 59 patients with various degrees of renal function impairment, including 14 patients with MM, were treated with bortezomib at escalating doses of 0.7, 1.0, and 1.3 mg/m^2^ [65]. The highest dose of this drug (1.3 mg/m^2^) was well tolerated in all groups of patients with RI, even in those requiring dialysis. Toxicities were reported to be generally mild, and no significant differences in bortezomib clearance were noticed regardless of creatinine clearance (CrCl). bortezomib 1.3 mg/m^2^ is well tolerated, and dose reductions are not necessary for patients with renal dysfunction [65]. Carfilzomib, another proteasome inhibitor but an irreversible one, appears to be effective in relapsed and refractory cases of MM; however, in the phase 2 trial by Siegel et al., 25% of patients experienced mild to moderate elevations in serum creatinine, questioning its usefulness in RF [66,67].

IMiDs such as thalidomide and lenalidomide, through a complex mechanism of action, interrupt the growth of malignant plasma cells and disrupt the interaction between myeloma cells and BM stromal cells [68,69]. The clearance of thalidomide is not dependent on renal function since this drug undergoes spontaneous hydrolysis in plasma; however, it is found to be associated with hyperkalemia in RF; therefore, the recommended dose of thalidomide is 50-100 mg/day for a patient with GFR less than 50 mL/min [70]. On the other hand, lenalidomide’s clearance is based on renal function and since it can cause myelosuppression, dose reduction is recommended based on GFR value and dialysis dependency [71]. Chen et al. conducted a study in 2007 where a single 25 mg dose of lenalidomide was administered in 30 patients with different renal functional status based on CrCl, and it was found that the mean urinary recovery of unchanged lenalidomide declined from 84% in patients with normal renal function (CrCl> 80 mL/min) to 38% and 43% in patients with moderate (CrCl 30-49 mL/min), and severe impairment (CrCl or <30 mL/min) [72]. Venous thromboembolic disease can be a potential drawback with high-dose dexamethasone regimens combined with IMiDs, particularly if there are other predisposing factors for venous thromboembolism, such as nephrosis, hypoalbuminemia, or concomitant erythropoietin use; therefore, empirical use of anticoagulation should be considered in those patients at highest risk [14]. Comparing conventional chemotherapy with novel agents, in 2010, Roussou et al. conducted a study on 96 patients with newly diagnosed MM with RI, where RI was defined as CrCl less than 50 mL/min, and in that study, major renal response (CRrenal+PRrenal) was found in 59%, 79%, and 94% of those patients treated with conventional chemotherapy, IMiDs, and bortezomib-based regimens, respectively [73]. The median time to response was 1.8 months for the patients treated with conventional chemotherapy as well as IMiDs whereas, it was only 0.69 months in the bortezomib group. Thus, it is concluded that bortezomib-based regimens may be the preferred treatment for newly diagnosed myeloma patients with RI [73]. Different studies on the efficacy and safety of different chemotherapeutic drugs are summarized in Table 2.

**Table 2:** Studies about different chemotherapeutic drugs for the treatment of MM with RF MM: multiple myeloma; RF: renal failure; ORR: overall response rate; CR: complete response; PR: partial response; nCR: near complete response; RI: renal impairment; CrCl: creatinine clearance; ImiDs: immunomodulatory drugs; MRR: major renal response

Study	Method	Result	Conclusion
Chanan-Khan et al., 2007 [58]	24 MM patients with dialysis-dependent advanced RF received bortezomib or bortezomib-based combination therapy.	ORR (CR + PR) = 75%. CR + nCR = 30%. Four patients recovered from dialysis.	Bortezomib or bortezomib-based regimens can be used in dialysis-dependent MM patients with manageable toxicities.
Leal et al., 2011 [65]	59 patients with various degrees of RI (including 14 MM patients) were treated with escalating doses of bortezomib (0.7, 1.0, and 1.3 mg/m^2^).	Highest dose of bortezomib (1.3 mg/m^2^) was well tolerated among all patients. Toxicities were mild. No significant difference in bortezomib clearance regardless of CrCl.	No dose reduction of bortezomib is necessary in MM patients with renal dysfunction.
Chen et al., 2007 [72]	30 patients with different renal functional statuses (based on CrCl) were given a single 25 mg dose of lenalidomide.	Mean urinary recovery of unchanged lenalidomide declined with CrCl value.	Lenalidomide dose reductions should be considered for patients with CrCl less than 50 mL/min.
Roussou et al., 2010 [73]	96 MM patients with dialysis-dependent RI were randomly treated with conventional chemotherapy (Group A), IMiDs (Group B), and bortezomib-based regimens (Group C).	MRR (CR+PR) was found in 94% in Group C and 59% and 79% in Group A and B, respectively. Median time to response (in months) was 1.8 for Group A as well as Group B and 0.69 for Group C.	Bortezomib-based regimens may be the preferred treatment for newly diagnosed MM patients with RI.

Autologous Hematopoietic Stem Cell Transplantation (Auto-HSCT)

RF, one of the worst complications of MM, may unfortunately prevent a patient from qualifying for HSCT, one of the most effective treatments of the disease [74]. In 2004, Lee et al. conducted a study where, 59 MM patients with dialysis-dependent RF were conditioned with high-dose melphalan (200 mg/m^2^) before auto-HSCT [64]. As per that study, dialysis duration less or equal to six months prior to the HSCT and pre-transplant CrCl more than 10 mL/min were significant for renal function recovery. For stem cell mobilization, plerixafor, has been approved in failed Neupogen mobilization [13]. Plerixafor is a selective antagonist of the CXC chemokine receptor 4, which reversibly inhibits the chemokine stromal cell-derived factor-1α [13].

There is no optimal conditioning regimen for MM with RF; however, the feasibility of reduced intensity conditioning has been studied in order to reduce the treatment-related mortality and graft-versus-host disease as well as to preserve antimyeloma activity [13]. For example, lesser dose of melphalan (140 mg/m^2^) has been associated with lesser toxicities although, it remains controversial whether high dose (200 mg/m^2^) of the drug makes any difference in disease response or survival in MM with RF [75,76]. As per the International Myeloma Working Group (IMWG) guidelines, melphalan at a dose of 140 mg/m^2^ can be used as conditioning for auto-HSCT in MM with RF (CrCl<60 mL/min) as well as for those on dialysis [71]. Scheid et al. conducted a study in 2013 where 827 patients of MM with RF were randomly administered with three cycles of VAD and PAD (bortezomib, Adriamycin, doxorubicin) followed by auto-HSCT and maintenance with thalidomide (50 mg) in VAD arm and bortezomib (1.3 mg) in PAD arm [77]. There was statistically significant better CR (p=0.01), OS at three years (p<0.001) and progression free survival rate at three years (p=0.004) in PAD arm concluding that in patients with newly diagnosed MM, bortezomib-containing treatment before and after the auto-HSCT can overcome the negative prognostic impact of RI.

Allogenic HSCT

Although severe RI is usually a contraindication to allogeneic SCT, there are studies supporting that a combined kidney and bone marrow transplantation (BMT) from an human leukocyte antigen (HLA) identical sibling donor with nonmyeloablative conditioning (cyclophosphamide, antithymocyte globulin, and thymic irradiation) along with post-transplant cyclosporine and donor leukocyte infusions can achieve renal allograft acceptance long term as well as excellent myeloma responses, even in the presence of donor marrow rejection and anti-donor alloresponses in vitro [13,14,78]. Patients with complete chimerizaton do not require post-transplant immunosuppressive therapy since the transplanted BM never rejects a kidney from the same donor [78].

Renal Transplantation

Occasionally, patients with myeloma-associated RF (particularly from LCDD and amyloid kidney) achieve a sustained complete hematologic response but still remain dialysis-dependent, and these patients are considered as renal transplant candidates regardless of whether HSCT is performed [14,79]. However, CR has to be achieved (especially in LCDD) at least three years before renal transplantation, otherwise, relapse can occur within 40 months [80]. Pre-transplant counseling should be done addressing the risks of kidney transplant that include MM relapse and disease progression (possibly as a result of the effects of immunosuppression), monoclonal Ig-mediated graft dysfunction, and infection [13,14,81].

## Conclusions

RF in MM is a medical emergency that requires prompt accurate diagnosis and management. Serum electrophoresis and immunofixation along with SFLC assay can accurately diagnose almost all cases. Renal biopsy should be considered early since it has diagnostic and prognostic value. While managing such cases, various causes should be addressed simultaneously. Dialysis should be considered in patients failing to respond with general measures. MM directed therapy should be started as soon as possible that includes chemotherapy and HSCT. As per the studies reviewed in this article, novel chemotherapeutic agents are considered to be superior to conventional drugs. Bortezomib, a proteasome inhibitor, can be effectively used in MM patients with renal dysfunction and no dose reduction is required. However, this is not true for other available drugs like melphalan, thalidomide, and lenalidomide, etc. Regarding auto-HSCT, pre-transplant CrCl more than 10 mL/min and dialysis duration less or equal to 6 months are important for renal function recovery. Although, there is no optimal conditioning regimen, Bortezomib-containing treatment before and after the auto-HSCT can overcome the negative prognostic impact of RIt. The idea of receiving simultaneous kidney and BMT from HLA identical sibling donors needs to be studied further. In patients with complete remission from MM for at least three years, kidney transplantation alone remains an option with its own risks of MM relapse and progression. Regarding SFLC removal, plasma exchange has no additional benefit over chemotherapy whereas, extended hemodialysis with a new generation HCO-HD has promising results and this technique is being studied in different trials. Successful RF management in MM patients remains a challenge in high-risk patients, necessitating additional evidence from future research. The need of the hour is for highly personalized treatment and collective decisions. Finally, we strongly feel that the link between MM and RF requires deep insight research studies to be conducted to develop a more structured and direct approach to diagnose, manage and prevent these conditions. However, up till now, the role of dialysis, chemotherapy and HSCT seems integral in the management of RF in MM patients.
